# Compliance With Daily Disposable Contact Lens Replacement Among Contact Lens Wearers: A Cross-Sectional Study

**DOI:** 10.7759/cureus.110288

**Published:** 2026-06-05

**Authors:** Ithar M Beshtawi, Mohammed Aljarousha

**Affiliations:** 1 Department of Optometry, Faculty of Medicine and Allied Medical Sciences, An-Najah National University, Nablus, PSE; 2 Department of Optometry, Management and Science University, Selangor, MYS

**Keywords:** compliance, daily disposable contact lenses, eye health equity, patient satisfaction, quality of life

## Abstract

Purpose

The main purpose of this study is to evaluate adherence to daily disposable contact lens (DDCL) replacement, identify factors contributing to non-compliance, and assess related risk behaviors.

Materials and methods

This cross-sectional study included 144 DDCL users, of whom 80 were female (55.56%). The mean age of participants was 27.80 ± 7.22 years. Participants completed a standardized questionnaire covering demographic characteristics, contact lens (CL) history, replacement habits, self-reported side effects, risky lens-wearing behaviors, and care instructions.

Results

Lens reuse was reported by 83 (57.64%) participants, and was significantly associated with gender, age, and wear duration (p < 0.05). Reuse was mainly driven by cost-saving in 35 (24.31%) participants and belief in reusability (24, 16.67%). Only 37 (25.69%) participants followed proper cleaning steps. Common risky behaviors included napping with lenses in 113 (78.47%) participants and skipping follow-up visits in 106 (73.61%) participants, both of which were significantly linked to reuse (p < 0.05). Only 36 (25.00%) participants believed daily replacement was essential, while 29 (20.14%) perceived no risk. Although 70 (48.61%) participants received verbal care instructions, 112 (77.78%) preferred written and video formats. Notably, 75 (52.08%) participants were unaware of the risks of reuse, contributing to non-compliance (p < 0.05).

Conclusions

Non-compliance was influenced by financial constraints, misconceptions about lens safety, lack of proper education, younger age, female gender, and limited lens experience. Improved, multimodal patient education is essential to promote safer practices and enhance ocular health outcomes.

## Introduction

Soft contact lenses (SCLs) are now widely prescribed and used globally [1], including in the Middle East [2,3] and Palestine [4,5]. These lenses are available in various types, with replacement schedules ranging from daily to annual use [1-4,6]. Global surveys on contact lens (CL) prescribing patterns show that daily disposable contact lenses (DDCLs) account for 39.00% of all fits, closely comparable to daily reusable lenses at 42.00% [1]. In Palestine, a recent study [4] found that 50.00% of users wore reusable lenses, while only 6.0% used DDCLs, likely due to the higher cost of disposables.

Research [7-11] has shown that frequent replacement of SCLs reduces deposits and contamination, improves ocular health, enhances clinical and visual performance [7,8], and increases overall satisfaction [9], compared to less frequent replacement. However, these benefits depend on consistent adherence to replacement schedules [9-11]. Unfortunately, compliance is often poor [10,11], which can lead to serious complications such as corneal inflammation, infections, and discomfort, which are leading causes of CL dropout [9]. In Palestine, a study [11] on CL care practices reported a 76.80% compliance rate but also found bacterial contamination in nearly 20.00% of users. Non-compliance was linked to poor hand hygiene, inadequate cleaning of lens cases, and water exposure during activities such as swimming, showering, or rinsing lenses with tap water [11].

DDCLs have grown in popularity [4,12], and eye care professionals (ECPs) often recommend them for users experiencing frequent lens deposits, difficulty with lens care, or discomfort [7,8,13]. These lenses offer several advantages, including reduced solution-related sensitivity [14], lower risk of lens case contamination, fewer ocular symptoms, and decreased complication rates [7,8,11-14]. However, these benefits depend on correct single-use behavior. Some users fail to replace their DDCLs daily [15,16], which may increase the risk of adverse events, including microbial keratitis. Previous studies have reported DDCL non-compliance rates ranging from 4.00% to 30.00% [15], with non-compliance often attributed to insufficient instruction, poor risk awareness, cost-related factors, or misunderstanding of replacement schedules. Some users may also misuse storage cases or cleaning solutions, despite DDCLs being designed for single use [10,11,15,16].

Although CL compliance has been studied internationally and in Palestine [10,11], most available evidence focuses on general CL wear or reusable lens care practices rather than DDCL-specific replacement behavior. In particular, there is limited evidence from Palestine and the wider Middle Eastern context regarding the frequency of DDCL reuse, the reasons users fail to replace lenses daily, and associated risky behaviors such as storage, cleaning, disinfection, and lens case use when DDCLs are reused. This gap is important because DDCLs are commonly perceived as safer and simpler than reusable lenses [7,8,13], yet their safety advantage may be reduced when users do not follow the intended single-use replacement schedule.

This study aimed to assess compliance with DDCL replacement among users in Palestine and to identify factors associated with non-compliance. It also examined associated lens care behaviors, particularly risky lens-use practices related to cleaning, disinfection, case hygiene, and storage in situations where DDCLs are reused. In addition, the study evaluated patient education regarding lens care and the risks of DDCL reuse. Given that improper or extended CL use may be associated with discomfort, blurred vision, corneal inflammation, and more serious complications such as microbial keratitis, highlighting factors associated with unsafe DDCL practices, this research may support targeted patient education and preventive eye care strategies aimed at reducing avoidable CL-related complications.

## Materials and methods

This cross-sectional analytical study included current users of DDCLs who were aged over 18 years, had worn DDCLs for at least six months, used no other CL type, and provided signed informed consent. Participants were excluded if they were younger than 18 years, had worn DDCLs for less than six months, used DDCLs in combination with other CL types, declined consent, were ineligible after screening, or submitted incomplete questionnaires. Participants were recruited using convenience sampling through social media announcements and flyers placed in optometry clinics across multiple cities in the West Bank, Palestine. Data were collected between August 2025 and March 2026.

A total of 182 individuals responded to the study invitation. Of these, 161 were eligible to participate, and 144 completed the questionnaire and were included in the final analysis. The eligibility rate was 88.46%, and the completion rate among eligible participants was 89.44%. Incomplete questionnaires were excluded.

As there was no prior data on the prevalence of non-compliance with DDCL replacement in this population, no formal sample size calculation was performed. Ultimately, 144 participants were enrolled based on their accessibility during the study period. Eligible participants completed a structured questionnaire (Appendix 1) during interviews conducted by a trained researcher. The questionnaire was developed by the researchers based on previously published studies and concepts related to CL compliance [10,15,16]. The questionnaire items were adapted and modified to suit the objectives and target population of the present study. The questionnaire was reviewed by experts for relevance, clarity, and content validity. The final version underwent pilot testing among 15 DDCL users to assess item clarity, wording, and feasibility of administration, and was subsequently refined. The internal consistency of the risky lens-use behavior items was assessed using Cronbach’s alpha and demonstrated good reliability (Cronbach’s alpha = 0.86). Responses from the pilot group were excluded from the final data analysis.

The questionnaire collected demographic information (age, gender, and education level) and detailed participants’ DDCL use history, including lens design, purchase source, duration of wear, daily wear time, and prior use of other CL types. Additionally, participants were asked to rate the importance of daily lens replacement (very important, moderately important, important, or not important) and report any adverse symptoms such as discomfort, blurred vision, corneal irritation, vision loss, or no symptoms. They were also surveyed about risky behaviors related to CL use, including hand hygiene before handling, showering or swimming with lenses, sleeping or napping while wearing lenses, sharing lenses, and missing follow-up appointments.

In this study, compliance was defined as discarding lenses each night and using a new pair every morning. Using the same lenses for more than one day was considered non-compliance [10,11,16]. Participants who reused lenses were asked to explain their reasons, such as cost-saving, belief in lens durability, running out of lenses, lack of awareness of risks, or receiving misleading recommendations. They were also asked to report how many days they reused lenses and whether they experienced any pain or discomfort.

Participants who reused lenses were further questioned about lens care practices during reuse. This included the type of solution used (e.g., multipurpose solution, sterile water, or leftover blister pack solution), storage method (e.g., lens case, original blister pack, or other containers), and whether they rubbed or rinsed the lenses before storage. At the end of the survey, participants were asked whether they had received instructions on DDCL care and the risks of reuse. They were also asked about the format of these instructions (verbal, written, video, or a combination) and their preferred format for future guidance.

Ethics statement

The study adhered to the ethical principles of the Declaration of Helsinki and was approved by the Institutional Review Board (IRB) of An-Najah National University, Nablus, Palestine (Ref: Med. Aug 2025/09). All participants gave informed consent before participating.

Statistical analysis

Data were analyzed using IBM SPSS Statistics for Windows, Version 20.00 (Released 2011; IBM Corp., Armonk, NY, USA). Descriptive statistics included means and standard deviations (SDs) for continuous variables and frequencies and percentages for categorical variables. Since the data were not normally distributed, non-parametric tests were used. Associations between categorical variables were assessed using Chi-square (χ²) tests; Fisher’s exact test was applied when expected cell counts were below five. A multivariable logistic regression model was used to identify independent predictors of DDCL replacement non-compliance. Variables found to be significant in bivariate analysis were included as covariates. The dependent variable was DDCL replacement non-compliance, defined as reuse of DDCLs beyond one day. Adjusted odds ratios (ORs) and 95% confidence intervals (CIs) were calculated. A p-value ≤ 0.05 was considered statistically significant.

## Results

Table 1 presents the demographic characteristics of the study participants. A total of 144 DDCL users participated in the study, including 80 (55.56%) females and 64 (44.44%) males. The average age of participants was 27.80 ± 7.22 years, ranging from 18 to 36 years. Most participants were aged 18-23 years (57, 39.58%), had undergraduate education levels (69, 47.92%), and reported low income levels (68, 47.22%).

**Table 1:** Demographic characteristics of the study participants (N = 144). The data has been represented as frequency (N) and percentage (%).

Variable	Category	Number (%)
Gender	Male	64 (44.44%)
	Female	80 (55.56%)
Age Group (years)	18-23	57 (39.58%)
	24-29	45 (31.25%)
	30-36	42 (29.17%)
Educational Level	High school	45 (31.25%)
	Undergraduate	69 (47.92%)
	Postgraduate	30 (20.83%)
Income Level	Low	68 (47.22%)
	Moderate	52 (36.11%)
	High	24 (16.67%)

Table 2 summarizes the characteristics of CL usage among the study participants. Spherical DDCLs were the most commonly used lens design, reported by 77 (53.47%) participants, followed by toric lenses used by 34 (23.61%) participants. With regard to the duration of CL use, 71 (49.31%) of the participants had been wearing CLs for 7-12 months, while 47 (32.64%) had used them for less than six months. Most participants purchased their lenses from optometry practices (102, 70.83%), whereas only seven (4.86%) obtained them through the internet. Regarding previous lens experience, 78 (54.17%) participants had previously used other types of CLs, most commonly monthly replacement SCLs (37, 25.69%). The majority of participants, 115 (79.86%), reported wearing lenses for two to four days per week, and 58 (40.28%) wore their lenses for five to eight hours a day.

**Table 2:** Contact lenses usage details of the study participants (N = 144). The data has been represented as frequency (N) and percentage (%). CLs: contact lenses; RGP: rigid gas permeable; SCLs: soft contact lenses; h: hours.

CL Usage Details	Number (%)
CL Brand Name	
Bausch & Lomb Soflens	28 (19.44%)
CooperVision Proclear 1-Day	27 (18.75%)
Morning-Q	5 (3.47%)
Acuvue Oasys 1-Day	7 (4.86%)
CooperVision Myday	11 (7.64%)
CooperVision Biomedics 1-day extra	9 (6.25%)
Daysoft SILK	6 (4.17%)
Alcon-Dailies Aqua Comfort	17 (11.81%)
Alcon-Focus Dailies CL	13 (9.03%)
Johnson & Johnson Vision Care 1-Day Acuvue	21 (14.58%)
Current CL Design	
Sphere	77 (53.47%)
Toric	34 (23.61%)
Progressive	6 (4.17%)
Bifocal	12 (8.33%)
Cosmetics	15 (10.42%)
Duration of CL Wear	
1-6 months	47 (32.64%)
7-12 months	71 (49.31%)
2-5 years	23 (15.97%)
6-10 years	3 (2.08%)
CL Purchase Source	
Optometry practice	102 (70.83%)
Pharmacy	14 (9.72%)
Internet	7 (4.86%)
Cosmetics store	21 (14.58%)
Other Types Used Before	
RGP	7 (4.86%)
Weekly replacement SCL	13 (9.03%)
Monthly replacement SCL	37 (25.69%)
Yearly replacement SCL	21 (14.58%)
None	66 (45.83%)
Days per Week of Lens Wear	
1 day	18 (12.50%)
2-4 days	115 (79.86%)
5-7 days	11 (7.64%)
Hours per Day of Lens Wear	
1-4 h	23 (15.97%)
5-8 h	58 (40.28%)
9-12 h	45 (31.25%)
More than 12 h	18 (12.50%)

Regarding compliance with recommended lens replacement practices, only 61 (42.36%) participants reported discarding their lenses after a single use, as recommended. The remaining 83 (57.64%) acknowledged reusing their lenses on subsequent days and were therefore classified as non-compliant. When participants were asked about the importance of daily lens replacement, 36 (25.00%) stated it was very important, 49 (34.03%) considered it somewhat important, 41 (28.47%) said it was important, and 18 (12.50%) felt it was not important (Table 3).

**Table 3:** Compliance, symptoms, and educational factors among DDCL users (n = 144). Data are presented as frequency (N) and percentage (%). DDCLs: daily disposable contact lenses; CLs: contact lenses.

Variable	Category	N (%)
Compliance with lens replacement	Compliant (single use only)	61 (42.36%)
	Non-compliant (lens reuse)	83 (57.64%)
Importance of daily replacement	Very important	36 (25.00%)
	Somewhat important	49 (34.03%)
	Important	41 (28.47%)
	Not important	18 (12.50%)
Instruction format received	Verbal	70 (48.61%)
	Written	38 (26.39%)
	Video	15 (10.42%)
	Combination	21 (14.58%)
Preferred instruction format	Verbal	7 (4.86%)
	Written	12 (8.33%)
	Video	13 (9.03%)
	Combination	112 (77.78%)
Education regarding the risks of reuse	No instructions about risks of reuse beyond one day	69 (47.92%)
	No information about the consequences of reuse	75 (52.08%)

Regarding patient education, participants were asked about both the format and content of the instructions they had received. In terms of instruction format, 70 (48.61%) participants reported receiving verbal guidance, 38 (26.39%) received written information, 15 (10.42%) watched video instructions, and 21 (14.58%) received a combination of formats. Notably, 112 (77.78%) expressed a preference for receiving lens care instructions through a combination of written and video materials. Regarding education specifically related to lens reuse, 75 (52.08%) stated they had received no information specifically about the potential consequences of reuse, while 69 (47.92%) participants reported receiving instructions about the risks of reusing lenses beyond one day (Table 3).

Among the 83 participants who reused their lenses, 11 (13.25%) did so for one additional day, 28 (33.73%) reused them for two to four days, 37 (44.58%) for five to seven days, and seven (8.43%) for longer than a week (Table 4). Participants who reused their lenses cited several reasons for doing so. The most common reason was cost-saving, reported by 35 (42.17%). A further 24 (28.92%) believed that lenses were safe to reuse, while 10 (12.05%) had simply run out of lenses. Other reasons included a perceived lack of risk in eight (9.64%) and recommendations from others suggesting that reuse was acceptable in six (7.23%) (Table 4).

**Table 4:** Reuse patterns, reasons for reuse, lens care practices, and symptoms among non-compliant DDCL users (n = 83). Data are presented as frequency and percentage (%). DDCL: daily disposable contact lens; CL: contact lens.

Variable	Category	N (%)
Duration of lens reuse	1 additional day	11 (13.25%)
	2-4 days	28 (33.73%)
	5-7 days	37 (44.58%)
	>1 week	7 (8.43%)
Reasons for lens reuse	Cost-saving	35 (42.17%)
	Belief lenses are reusable	24 (28.92%)
	Ran out of lenses	10 (12.05%)
	No perceived risk	8 (9.64%)
	Advice from others	6 (7.23%)
Cleaning solution used during reuse	Multipurpose solution	37 (44.58%)
	Sterile water	28 (33.73%)
	Residual blister solution	14 (16.87%)
	Tap water	4 (4.82%)
Lens rubbing/rinsing before storage	Rubbed/rinsed lenses before storage	44 (53.01%)
	Did not rub/rinse lenses	39 (46.99%)
Lens storage method	CL case	37 (44.58%)
	Original blister pack	27 (32.53%)
	Other containers	17 (20.48%)
	Other methods	2 (2.41%)
Symptoms associated with non-replacement	Discomfort	34 (40.96%)
	Blurred vision	21 (25.30%)
	Corneal inflammation	14 (16.87%)
	Vision loss	1 (1.20%)
	No symptoms	16 (19.28%)

Among participants who reused their lenses, several unsafe lens care practices were identified. Regarding cleaning practices, 37 (44.58%) used a multipurpose solution for cleaning, 28 (33.73%) used sterile water, 14 (16.87%) used leftover solution from blister packs, and four (4.82%) used tap water. Regarding pre-storage handling practices, 39 (46.99%) participants reported that they did not rub or rinse the lenses before storing them. Storage methods varied: 37 (44.58%) used CL cases, 27 (32.53%) stored lenses in the original blister pack, 17 (20.48%) used available containers such as glasses or cups, and two (2.41%) reported using other methods (Table 4). Participants also reported symptoms associated with failure to replace DDCLs daily. Discomfort was the most common symptom, reported by 34 (40.96%), followed by blurred vision in 21 (25.30%) and corneal inflammation in 14 (16.87%). A small number, one (1.20%), reported loss of vision, while 16 (19.28%) did not report any side effects (Table 4).

**Figure 1:**
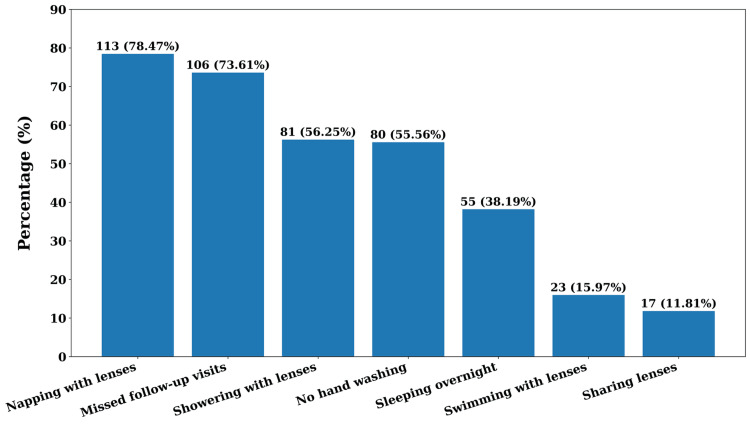
Risky behaviors reported among daily disposable contact lens users (N = 144). Data are presented as frequency (N) and percentage (%).

Participants also engaged in various risky behaviors while wearing their lenses. The most frequently reported non-compliant behavior was napping while wearing lenses, cited by 113 (78.47%) participants. This was followed by missing scheduled follow-up visits in 106 (73.61%) participants, showering while wearing lenses in 81 (56.25%) participants, handling lenses without washing hands in 80 (55.55%) participants, sleeping overnight with lenses in 55 (38.19%) participants, swimming while wearing lenses in 23 (15.97%) participants, and sharing lenses in 17 (11.80%) participants (Figure 1).

Bivariate analysis using Chi-square tests demonstrated significant associations between non-compliance and several participant characteristics (Table 5). Female participants were significantly more likely to report lens reuse compared to males (χ²(1, N = 144) = 13.23, p < 0.001). Age group was also significantly associated with non-compliance. The highest prevalence was observed among participants aged 24-29 years (χ²(2, N = 144) = 11.73, p < 0.001). Significant associations were additionally identified for prior CL experience (χ²(1, N = 144) = 7.62, p < 0.001), duration of CL wear (χ²(3, N = 144) = 9.80, p = 0.02), and days per week of lens wear (χ²(2, N = 144) = 8.12, p = 0.01). Participants who had worn CLs for 7-12 months and those who wore lenses two to four days per week showed higher rates of non-compliance. Furthermore, lack of education regarding the risks and consequences of lens reuse was significantly associated with non-compliance (χ²(1, N = 144) = 10.23, p = 0.03). Risky behaviors were also associated with lens reuse, as missing follow-up visits (χ²(1, N = 144) = 10.79, p = 0.04) and sleeping overnight while wearing lenses (χ²(1, N = 144) = 18.78, p = 0.03) were both significantly linked to non-compliance. In contrast, educational level and income level were not significantly associated with compliance behavior (p > 0.05).

**Table 5:** Bivariate analysis of factors associated with non-compliance to daily disposable contact lens replacement among study participants (N = 144). Data are presented as frequency (N) and percentage (%). *Significant at p < 0.05; χ² denotes Chi-square test statistic. CLs: contact lenses; df: degrees of freedom.

Variable	Category	Compliant	Non-compliant	Total	χ² (df)	p-value
		N (%)	N (%)	N (%)		
Gender	Male	45 (70.31%)	19 (29.68%)	64 (44.44%)	χ²(1) = 13.23	<0.001*
	Female	16 (20.0%)	64 (80.00%)	80 (55.56%)		
Age group	18-23	43 (75.44%)	14 (24.56%)	57 (39.58%)	χ²(2) = 11.73	<0.001*
	24-29	6 (13.33%)	39 (86.67%)	45 (31.25%)		
	30-36	12 (28.57%)	30 (71.43%)	42 (29.17%)		
Educational level	High school	18 (40.00%)	27 (60.00%)	45 (31.25%)	χ²(2) = 0.77	0.68
	Undergraduate	30 (43.48%)	39 (56.52%)	69 (47.92%)		
	Postgraduate	13 (43.33%)	17 (56.67%)	30 (20.83%)		
Income	Low	28 (41.18%)	40 (58.82%)	68 (47.22%)	χ²(2) = 0.71	0.70
	Moderate	20 (38.46%)	32 (61.54%)	52 (36.11%)		
	High	13 (54.17%)	11 (45.83%)	24 (16.67%)		
Prior lens experience	Yes	43 (53.75%)	37 (46.25%)	80 (55.56%)	χ²(1) = 7.62	0.01*
	No	18 (28.13%)	46 (71.88%)	64 (44.44%)		
Duration of contact lens wear	1-6 months	38 (80.85%)	9 (19.15%)	47 (32.64%)	χ²(3) = 9.80	0.02*
	7-12 months	13 (18.57%)	57 (81.43%)	70 (48.61%)		
	2-5 years	6 (28.57%)	15 (71.43%)	21 (14.58%)		
	6-10 years	4 (66.67%)	2 (33.33%)	6 (4.17%)		
Days per week of lens wear	1 day	14 (77.78%)	4 (22.22%)	18 (12.50%)	χ²(2) = 8.12	0.02*
	2-4 days	30 (26.09%)	85 (73.91%)	115 (79.86%)		
	5-7 days	6 (54.55%)	5 (45.45%)	11 (7.64%)		
Instructions regarding the risks of lens reuse	Received instructions	19 (27.54%)	50 (72.46%)	69 (47.92%)	χ²(1) = 10.23	0.03*
	Did not receive instructions	42 (56.00%)	33 (44.00%)	75 (52.08%)		
Risky Behaviors						
Napping while wearing lenses	Yes	40 (35.40%)	73 (64.60%)	113 (78.47%)	χ²(1) = 2.41	0.12
	No	21 (67.74%)	10 (32.26%)	31 (21.53%)		
Missed follow-up visits	Yes	32 (30.19%)	74 (69.81%)	106 (73.61%)	χ²(1) = 10.79	0.04*
	No	29 (76.32%)	9 (23.68%)	38 (26.39%)		
Showering while wearing lenses	Yes	29 (35.80%)	52 (64.20%)	81 (56.25%)	χ²(1) = 1.96	0.16
	No	32 (50.79%)	31 (49.21%)	63 (43.75%)		
Handling lenses without washing hands	Yes	30 (37.50%)	50 (62.50%)	80 (55.56%)	χ²(1) = 1.02	0.31
	No	31 (48.44%)	33 (51.56%)	64 (44.44%)		
Sleeping overnight with lenses	Yes	14 (25.45%)	41 (74.55%)	55 (38.19%)	χ²(1) = 18.78	0.04*
	No	47 (52.81%)	42 (47.19%)	89 (61.81%)		
Swimming while wearing lenses	Yes	7 (30.43%)	16 (69.57%)	23 (15.97%)	χ²(1) = 1.87	0.17
	No	54 (44.63%)	67 (55.37%)	121 (84.03%)		
Sharing lenses with others	Yes	5 (29.41%)	12 (70.59%)	17 (11.81%)	χ²(1) = 1.54	0.21
	No	56 (44.09%)	71 (55.91%)	127 (88.19%)		

Multivariable logistic regression analysis was performed to identify factors independently associated with non-compliance with DDCL replacement (Table 6). The model included gender, age group, educational level, income, prior CL experience, duration of CL wear, and days per week of lens wear. After adjustment, prior CL experience remained significantly associated with non-compliance, as participants without previous lens experience were more likely to be non-compliant (adjusted odds ratio (aOR) = 2.45; 95% CI: 1.30-4.62; p < 0.001). Gender also remained a significant predictor, with males being less likely to exhibit non-compliance compared to females (aOR = 0.38; 95% CI: 0.18-0.80; p = 0.01). Participants aged 24-29 years were significantly more likely to be non-compliant compared to those aged 18-23 years (aOR = 3.12; 95% CI: 1.42-6.85; p < 0.001). In contrast, educational level (aOR = 1.12; 95% CI: 0.66-1.89; p = 0.68), income (aOR = 0.90; 95% CI: 0.52-1.56; p = 0.70), duration of CL wear (aOR = 1.45; 95% CI: 0.75-2.81; p = 0.27), and days per week of lens wear (aOR = 1.37; 95% CI: 0.73-2.57; p = 0.33) were not independently associated with non-compliance after adjustment.

**Table 6:** Multivariable logistic regression analysis of factors associated with non-compliance to daily disposable contact lens replacement (N = 144). *Significant at p < 0.05. aOR: adjusted odds ratio; CI: confidence interval.

Variable	aOR	95% CI	p-value
Gender	0.38	0.18-0.80	0.01*
Age group	3.12	1.42-6.85	<0.001*
Educational level	1.12	0.66-1.89	0.68
Income	0.90	0.52-1.56	0.70
Prior contact lens experience	2.45	1.30-4.62	0.01*
Duration of contact lens wear	1.45	0.75-2.81	0.27
Days per week of lens wear	1.37	0.73-2.57	0.33

## Discussion

Although DDCLs are designed to reduce complications associated with CL wear [8,13] by eliminating the need for cleaning and storage [7,13], patients may still fail to adhere to the recommended replacement schedule and proper care behaviors [15,16]. This is particularly concerning, as many DDCL users may not receive instructions on appropriate care practices, especially those relevant to lens reuse. The findings of this study offer important insights into DDCL replacement compliance, lens care behaviors among those who reuse lenses, the reasons behind such misuse from the patients’ perspectives, and the impact of receiving proper care instructions on improving compliance. From a broader public health perspective, these findings are relevant to Sustainable Development Goal 3: Good Health and Well-Being, as improving DDCL compliance through patient education, appropriate prescribing, and regular follow-up may help reduce preventable CL-related complications and promote safer use of medical devices [9,13].

In this study, DDCL use was more common among younger and female participants. This aligns with previous research suggesting that younger users prefer DDCLs due to their convenience, low maintenance, and suitability for active lifestyles [12,15,16]. It also supports earlier findings that DDCLs are more frequently used by females [15,16], who often choose CL for cosmetic reasons [1,4,5]. However, this finding contrasts with other studies reporting higher usage rates among males [12]. The data also revealed that more than half of the participants used spherical DDCLs, consistent with international prescribing trends [12]. This may reflect the wider use of DDCLs among individuals with spherical refractive errors and minimal astigmatism, as well as the higher cost and limited availability of more complex lens designs [12,15]. Furthermore, more than half of participants had previous experience with other types of CL before switching to DDCLs, suggesting a gradual transition toward DDCLs, as patients increasingly prioritize comfort, safety, and ease of use [7-9,13], despite the higher cost associated with this modality [17].

Although DDCLs are widely used [12,15] and have been associated with lower non-compliance rates compared to reusable lenses [13], this study found that more than half of participants reused their lenses, with a small proportion of non-compliant users extending reuse beyond one week. This rate is notably higher than in previous studies, such as one conducted in Italy, where only 22.60% reported reuse [16], and another multicenter study in which 91.00% of participants adhered to the recommended daily replacement [15]. The high prevalence of DDCL reuse observed in the current study raises significant clinical concerns, as reusing lenses - particularly without proper cleaning or disinfection - substantially increases the risk of ocular complications [18].

The analysis identified several factors associated with DDCL non-compliance. In the multivariable logistic regression model, prior CL experience emerged as an important independent predictor, with less experienced lens users being more than twice as likely to be non-compliant with DDCL replacement. Gender also remained significant, with males being less likely to report non-compliant behavior compared with females, although this finding contrasts with a previous report [11], suggesting better compliance among women. Age was another important predictor, with participants aged 24-29 years showing higher odds of non-compliance than those aged 18-23 years. This may reflect financial pressures and behavioral patterns during the transition from student life to early career stages [10,16,17]. In addition, shorter duration of CL wear and intermittent weekly use may reflect misconceptions that occasional DDCL wear carries a lower risk and therefore justifies extending lens use. In contrast, educational level and income were not significantly associated with compliance, suggesting that general socio-economic indicators may be less influential than lens-wearing experience and specific behavioral habits [10]. These associations should be interpreted cautiously, because the cross-sectional design does not allow causal inference.

Cost-saving was the most frequently cited reason for reusing DDCLs. Financial constraints appear to drive some users to extend lens use beyond the recommended timeframe. In such cases, ECPs may consider recommending reusable lenses as a more cost-effective and safer alternative [17]. The second most common reason was a belief in the reusability of daily lenses, indicating gaps in patient knowledge or confusion with other lens types [16,19]. These findings are consistent with earlier studies [15,16] reporting non-adherence among younger individuals, often driven by financial limitations and a lack of understanding of the associated risks [10]. Some participants also reported that they reused lenses because they had run out of replacements, suggesting that practical interventions such as automated reminders or CL compliance apps could help reduce unintentional non-compliance [19].

A concerning finding was that more than half of the participants indicated they had not received education about the possible consequences associated with non-compliance. This may contribute to the underestimation of risk, particularly among users who believe that daily lenses can be safely reused or that intermittent wear reduces the likelihood of complications [10]. Although most participants had formal education, unsafe DDCL reuse and care practices remained frequent, indicating that general educational attainment does not necessarily translate into safe CL behavior. Therefore, structured CL-specific education should be provided at the initial consultation and reinforced during follow-up visits [10,16]. Digital tools, including virtual reminders and CL compliance applications, may also help reduce unintentional non-compliance [19].

Participants who reused DDCLs also reported unsafe cleaning and storage practices, including the use of multipurpose solutions, sterile water, residual blister solution, tap water, original blister packs, and non-sterile containers such as cups or glasses. Some participants also reported not rubbing or rinsing lenses before storage, although mechanical rubbing is important for reducing microbial contamination even when no-rub solutions are used [18-20]. These behaviors were reported in previous studies [10,11,19] and are clinically concerning because inappropriate cleaning and storage may increase the risk of lens contamination [11], ocular inflammation, microbial keratitis [18], and other ocular surface complications [15]. Additional risky behaviors, including napping while wearing lenses, showering while wearing lenses, and missing scheduled follow-up visits, were also frequent and were associated with higher rates of lens reuse [18].

While this study offers valuable insights into factors associated with compliance with DDCL replacement, several limitations should be acknowledged. First, the cross-sectional design limits causal inference between the identified factors and DDCL non-compliance. Second, convenience sampling through social media announcements and clinic-based advertisements may have introduced selection bias and may limit generalizability. Third, reliance on self-reported data may have introduced recall and social desirability bias. In addition, the absence of clinical assessments limited the ability to verify non-compliance or detect related ocular complications. Finally, the relatively small sample size may have reduced statistical precision.

Despite these limitations, the study provides useful preliminary data in an area with limited evidence. Future studies should include larger and more diverse samples, with formal sample size calculations based on prevalence estimates from this study. Longitudinal designs are also needed to monitor compliance over time and assess whether structured education, multimedia tools, mobile applications, and virtual reminders can improve DDCL replacement adherence and reduce unsafe care practices.

## Conclusions

This study identified a substantial level of non-compliance with DDCL replacement schedules and related care practices. The main factors associated with this behavior included cost-saving motives, misconceptions about the reusability of lenses, and insufficient knowledge of proper CL care. Despite the benefits of DDCLs, many users reported engaging in high-risk behaviors, including extended reuse, inadequate cleaning, and unsafe storage, which may be associated with ocular complications. A notable proportion of DDCL users lacked awareness of the potential risks associated with lens reuse and had not received clear, comprehensive instructions from their ECPs. Multivariable analysis identified limited prior lens experience, female gender, and being aged 24-29 years as independent factors associated with non-compliance.

These findings support the need for targeted educational interventions for groups with higher observed non-compliance. Overall, the results highlight the importance of comprehensive, multimodal patient education that extends beyond verbal guidance. Educational strategies should address proper lens replacement, hygiene, and the potential consequences of non-compliance. ECPs should prioritize regular follow-up appointments and provide consistent, risk-focused counseling to enhance patient understanding, support informed decision-making, and promote safer DDCL use.

## Appendices

This questionnaire aims to assess compliance with daily disposable contact lens (DDCL) replacement among contact lens users in Palestine. Participation is voluntary, and all responses will remain confidential.

Appendix 1

Study Questionnaire Used to Assess Compliance With Daily Disposable Contact Lens Replacement Among Contact Lens Wearers

**Table 7:** Questionnaire.

Do you agree to take part in this study? (Please select one option)
Yes
No
1. Participant Information
What is your gender? (Please select one option)
Male
Female
Other
What is your age group? (Please select one option)
18-23
24-29
30-36
What is your educational level? (Please select one option)
Illiterate
High school
Diploma
Undergraduate
Postgraduate
How would you describe your income level? (Please select one option)
Low
Moderate
High
What is the brand name of the contact lenses you currently use? (Please select one option)
Bausch & Lomb Soflens
CooperVision Proclear 1-Day
Morning-Q
Acuvue Oasys 1-Day
CooperVision Myday
CooperVision Biomedics 1day extra
Daysoft SILK
Alcon-Dailies aqua comfort
Alcon-Focus Dailies CL
Johnson & Johnson Vision Care 1-Day Acuvue
Other (please specify: ________)
What type of contact lens do you currently wear? (Please select one option)
Spherical
Toric
Progressive
Bifocal
Cosmetic
How long have you been wearing your current contact lenses? (Please select one option)
1-6 months
7-12 months
2-5 years
6–10 years
Have you previously used other types of contact lenses? (Please select one option)
Yes
No
If yes, what type(s) of contact lenses have you used before? (You may select more than one option)
Rigid gas permeable (RGP)
Weekly replacement soft CL
Monthly replacement soft CL
Yearly replacement soft CL
None
How many days per week do you wear contact lenses? (Please select one option)
1 day
2-4 days
5-7 days
How many hours per day do you wear contact lenses? (Please select one option)
1-4 h
5-8 h
9-12 h
>12 h
3. Lens Reuse Information
Do you replace lenses daily as recommended? (Please select one option)
Yes
No
If you reuse lenses, for how long do you usually reuse them? (Please select one option)
1 additional day
2-4 days
5-7 days
>1 week
What is the reason for reusing your lenses? (You may select more than one option)
Cost-saving
Ran out of lenses
No perceived risk
Belief lenses are reusable
Advice from others
4. Lens Care Practices
What cleaning solution do you use when reusing lenses? (You may select more than one option)
Multipurpose solution
Sterile water
Residual blister solution
Tap water
How do you store reused contact lenses? (You may select more than one option)
Contact lens case
Original blister pack
Other containers
Other methods
Do you wash your hands before handling contact lenses? (Please select one option)
Yes
No
Do you rub/rinse lenses before storing them? (Please select one option)
Yes
No
Do you take naps while wearing contact lenses? (Please select one option)
Yes
No
Do you sleep overnight while wearing contact lenses? (Please select one option)
Yes
No
Do you shower while wearing contact lenses? (Please select one option)
Yes
No
Do you swim while wearing contact lenses? (Please select one option)
Yes
No
Do you share your contact lenses with others? (Please select one option)
Yes
No
Have you missed any scheduled follow-up visits related to your contact lens use? (Please select one option)
Yes
No
5. Risks and Patient Education
Have you received information regarding the risks associated with lens reuse? (Please select one option)
Yes
No
How important do you consider the daily replacement of contact lenses? (Please select one option)
Very important
Somewhat important
Important
Not important
Did you receive instructions regarding the risks associated with reusing daily disposable contact lenses beyond one day? (Please select one option)
Yes
No
What symptoms/complications do you think may be associated with lens reuse? (You may select more than one option)
Discomfort
Blurred vision
Corneal inflammation
Vision loss
No symptoms
In what format did you receive lens-care instructions? (Please select one option)
Verbal
Written
Video
Combination
What is your preferred format for receiving lens care instructions? (Please select one option)
Verbal
Written
Video
Combination
Were the instructions provided sufficient in your opinion? (Please select one option)
Yes
No
